# Dietary restriction ameliorates TBI-induced phenotypes in *Drosophila melanogaster*

**DOI:** 10.1038/s41598-022-13128-x

**Published:** 2022-06-09

**Authors:** Rebecca Delventhal, Emily R. Wooder, Maylis Basturk, Mohima Sattar, Jonathan Lai, Danielle Bolton, Gayathri Muthukumar, Matthew Ulgherait, Mimi M. Shirasu-Hiza

**Affiliations:** 1 Department of Biology, Lake Forest College, Lake Forest, IL 60045 USA; 2 Department of Genetics and Development, Columbia University Irving Medical Center, New York, NY 10032 USA

**Keywords:** Neural ageing, Diseases of the nervous system, Neurodegeneration, Ageing

## Abstract

Traumatic brain injury (TBI) affects millions annually and is associated with long-term health decline. TBI also shares molecular and cellular hallmarks with neurodegenerative diseases (NDs), typically increasing in prevalence with age, and is a major risk factor for developing neurodegeneration later in life. While our understanding of genes and pathways that underlie neurotoxicity in specific NDs has advanced, we still lack a complete understanding of *early* molecular and physiological changes that drive neurodegeneration, particularly as an individual ages following a TBI. Recently *Drosophila* has been introduced as a model organism for studying closed-head TBI. In this paper, we deliver a TBI to flies early in adult life, and then measure molecular and physiological phenotypes at short-, mid-, and long-term timepoints following the injury. We aim to identify the timing of changes that contribute to neurodegeneration. Here we confirm prior work demonstrating a TBI-induced decline in lifespan, and present evidence of a progressive decline in locomotor function, robust acute and modest chronic neuroinflammation, and a late-onset increase in protein aggregation. We also present evidence of metabolic dysfunction, in the form of starvation sensitivity and decreased lipids, that persists beyond the immediate injury response, but does not differ long-term. An intervention of dietary restriction (DR) partially ameliorates some TBI-induced phenotypes, including lifespan and locomotor function, though it does not alter the pattern of starvation sensitivity of injured flies. In the future, molecular pathways identified as altered following TBI—particularly in the short-, or mid-term—could present potential therapeutic targets.

## Introduction

Traumatic brain injury (TBI) is a growing global health concern; it is estimated that nearly 70 million people sustain a TBI every year globally^1^. TBI is known to cause a wide range of acute and chronic neurological defects, including long-term conditions such as chronic traumatic encephalopathy (CTE), neuroendocrine, and metabolic disorders^2–4^. TBI is also a well-established risk factor for developing a neurodegenerative disease (ND), such as Parkinson’s (PD) or Alzheimer’s disease (AD), later in life^4–6^. TBI and ND patients exhibit many of the same hallmarks: cognitive deficits, degradation of neuronal tissues, and increased protein aggregation in the brain^2,7,8^. This suggests that TBI and NDs might share common molecular pathways that drive neurodegeneration. Thus, it is possible that studying changes that occur following TBI may uncover common mechanisms of neurodegeneration.

Many molecular pathologies have been identified in multiple types of NDs and are also often observed with aging. Most NDs share a common molecular pathology of toxic protein aggregates, although the location and identity of the protein aggregates vary^8,9^. While much research has focused on the mechanisms underlying neurotoxicity of protein aggregates, the mechanisms that drive their accumulation in sporadic NDs are less well-understood, and their causal role has been debated^10,11^. Innate immune activation in the brain, neuroinflammation, has been suggested to play a role in the pathogenesis of a variety of NDs, in part through improper activation of microglia leading to neuronal damage^12,13^. TBI has also been demonstrated to result in long-term neuroinflammation, possibly contributing to neurodegeneration^14^. Oxidative stress and mitochondrial dysfunction are implicated in monogenic causes of Parkinson’s Disease, such as *Pink* and *Parkin* mutations, but also appear to be features found in other forms of ND^15–18^. While these common features of protein aggregates, neuroinflammation, and oxidative stress have been observed in many ND models, the causality is not always clear.

Metabolic dysfunction has recently been identified as both a symptom of, and potential contributing factor to, neurodegenerative disease^19–22^. Obesity is associated with higher incidence of dementia later in life^23^, and diabetes mellitus increases the risk of developing AD by 65%^24^ and occurs with a higher incidence in Huntington’s Disease patients^25^. Through years of animal model research, aberrant insulin signaling has been identified as playing a key role in pathogenesis of NDs^26–29^. Interestingly, unexplained weight *loss* is also a common feature in early and late stages of PD^30^ and is also considered a predictor of dementia and AD in patients with mild cognitive impairment^31^. Global alterations to brain metabolism, including hypometabolism (decreased glucose consumption in the brain) are associated with neurodegeneration^32,33^. TBI is associated with acute increase in glycolysis, followed by impaired metabolism for several days in mouse models^34,35^. The most critical metabolic changes and exactly how they may contribute to neurodegeneration, in particular, following TBI, remain unclear^15,36^.

Recent research has established *Drosophila* as a model organism for closed-head TBI using a few different methods of inflicting the injury^37–44^. In this paper, we adapted the approach first published by Katzenberger et al.^37^ in which a group of flies are placed in an empty vial attached to a spring that is deflected and released to impact a hard surface, delivering the injury in what is termed a “high-impact trauma” (HIT) device. Others have developed methods that injure smaller groups of flies in a bead mill homogenizer^41^ or injure individual flies in a collar with a piezoelectric actuator^39,45^ or with pressurized gas^42^. Through this work, it has been found that TBI-inflicted *Drosophila* exhibit acute ataxia, circadian arrhythmicity, structural damage to neurons and brain tissue, decreased lifespan, acute induction of immune gene expression, as well as expression of other pathways, such as antioxidant, metabolic, and unfolded protein response^37,39–41,46–48^. Another study examined the modification of genetic model of amyotrophic lateral sclerosis (ALS) by TBI, which further worsened locomotor ability in the ALS model and induced stress granule accumulation containing protein aggregates^49^.

Most previous work on TBI in *Drosophila* has focused on the acute or short-term injury responses, on the timescale of hours or days post-TBI. We were interested in studying the long-term consequences of TBI, especially those that phenocopy features of ND, because changes that occur prior to others could suggest a causal relationship. We hypothesized that describing the timing of physiological and molecular perturbations might identify potential targets and optimal times for intervention. Here we measured outcomes at multiple times post-TBI, including a long-term timepoint (4 weeks post-TBI) just before a significant portion of the injured population starts dying. We also found that dietary restriction, or the limitation of dietary protein to the extent that it extends *Drosophila* lifespan, ameliorates some TBI-induced pathogenic phenotypes. This suggests that pathways implicated in aging may also be perturbed following TBI.

## Results

We set out to examine physiological and molecular consequences of TBI that are also common symptoms of NDs. We measured these at multiple timepoints following the injury, primarily 48 h (short-term), 2 weeks (mid-term), and 4 weeks (long-term), to understand when different phenotypes may appear and disappear following the injury. In all experiments, we injured flies at 5–7 days of age (post-eclosion) using a paradigm adapted from Katzenberger et al., in which 40–45 flies are transferred without anesthesia to an empty vial, which is then attached to a metal spring, pulled back to a rod marking a controlled angle of deflection, then released to impact a plastic pad. Approximately 15–50% of flies die within 24–48 h (see supplemental data files for mortality indices for each dataset).

### TBI leads to decreased lifespan and progressive decline in locomotor function

**Figure 1:**
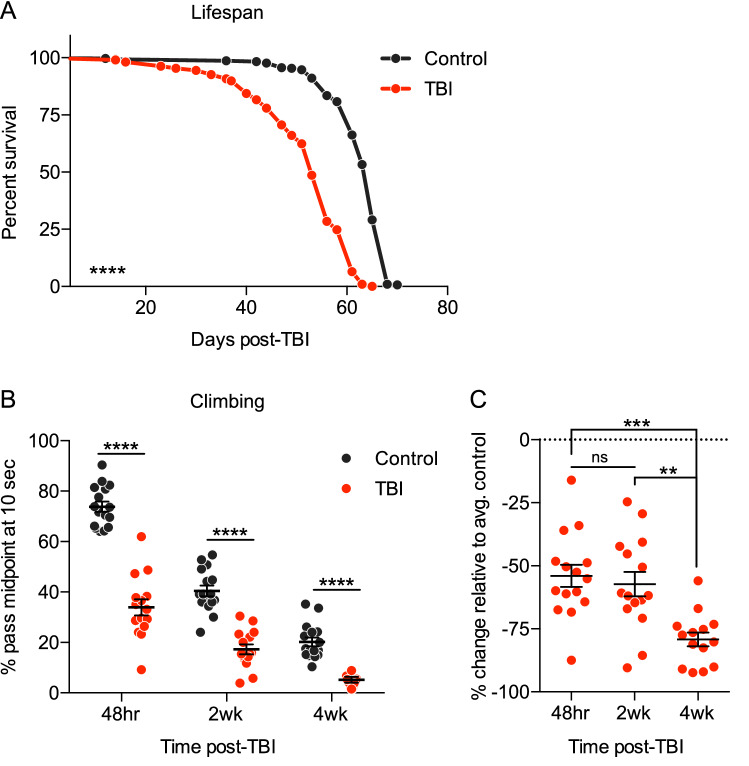
Traumatic Brain Injury (TBI) induces long-term physiological neurodegenerative phenotypes. TBI-treated flies = red; age-matched, uninjured control flies = dark grey. (**A**) TBI-treated flies exhibit significantly decreased lifespan, as previously reported^37,41^. Acute death in the first 48 h following injury is excluded. One of three independent repeat lifespan experiments is shown (sample size of 110–300 flies per condition). *p* < 0.0001 according to both Log-rank and Gehan-Breslow-Wilcoxon survival comparison tests. (**B**) TBI-treated flies exhibit significantly decreased climbing ability at multiple timepoints following the injury: 48 h, 2 weeks, and 4 weeks. Climbing ability is determined as the percent of flies past the midpoint of the climbing vial after 10 s of climbing (at least 14 groups of 20–25 flies each). TBI compared to control at each timepoint is *p* < 0.0001 according to two-way ANOVA, with Sidak’s multiple comparison correction. One outlier was identified and removed from 4 weeks TBI dataset according to ROUT method (Q = 1%). (**C**) TBI-treated flies display a larger decrease in climbing ability at 4 weeks post-TBI, calculated as percent change relative to average of age-matched, uninjured controls, than at 48 h and 2 weeks post-TBI. 48 h compared to 4 weeks: *p* < 0.001, 2 weeks compared to 4 weeks: *p* < 0.01; 48 h compared to 2 weeks: ns, according to a one-way ANOVA, with Tukey’s multiple comparisons test.

We first examined TBI-treated *Drosophila* for two common, long-term consequences of neurodegeneration: decreased lifespan and locomotor decline. Consistent with previously published findings^37,41^, TBI induced an ~ 18% decrease in lifespan, with a median survival of 53 days, compared to 65 days for uninjured control flies (Fig. 1A). We measured the locomotor ability of injured flies compared to age-matched, uninjured controls, using a climbing assay. We found that injured flies displayed decreased climbing ability relative to age-matched controls at every timepoint, short- to long-term, following the injury (Fig. 1B). This is consistent with recent studies showing long-term climbing deficits weeks following mild TBI, using a different injury method^50,51^. Moreover, post-injury defects in climbing ability worsened with age, as 4 week post-TBI flies displayed a significantly larger reduction (~ 75%) in climbing relative to the average climbing of controls of the same age, compared to 48 h and 2 week (~ 50%) post-TBI flies, relative to the average of their age-matched controls (Fig. 1C). Climbing ability also decreased with age in controls, as expected. Together, these results suggest that TBI has long-term effects on healthspan and lifespan in *Drosophila*, consistent with previous work.

### TBI leads to long-term accumulation of protein aggregates and increased immune gene expression in the head

**Figure 2:**
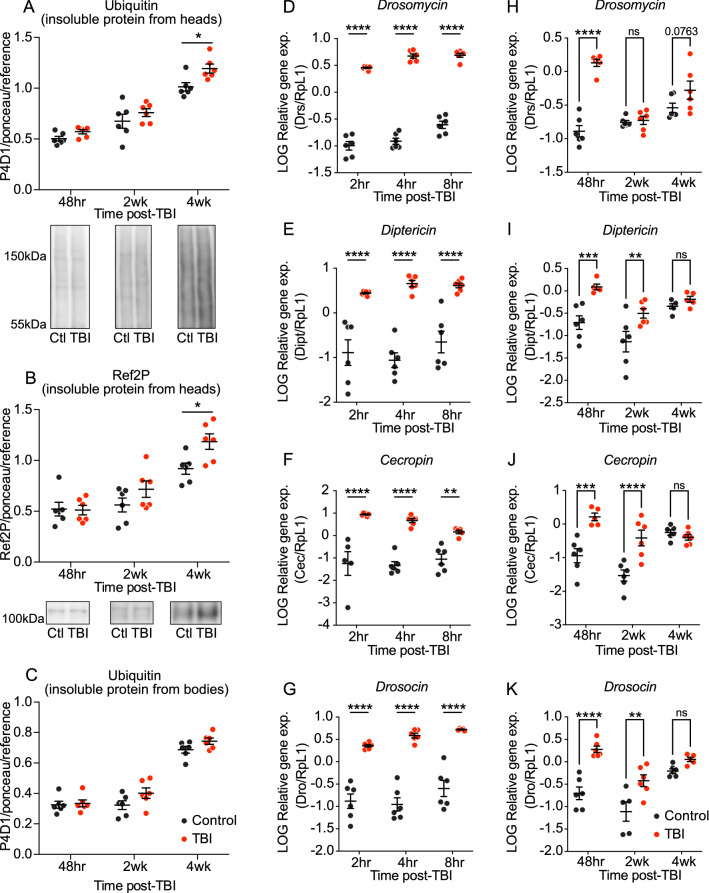
TBI induces long-term molecular hallmarks of neurodegeneration. TBI-treated flies = red; age-matched, uninjured control flies = dark grey. Insoluble protein extracted from heads of TBI-treated flies contains increased (**A**) mono- and poly-ubiquitinated protein between ~ 40 and 150 kDa, measured by P4D1 antibody, and (**B**) Ref2P, at 4 weeks post-TBI only, when compared to age-matched control flies. Insoluble protein extracted from bodies does not display a significant increase in (**C**) ubiquitinated protein. Six replicates of 50 heads each, or 20 bodies each, were extracted at each timepoint. TBI and control samples within each timepoint were run on the same western blot, normalized to total protein between 37 and ~ 200 kDa via Ponceau stain, and then normalized to a standard reference protein sample of mixed old and young flies, which was included on each western blot. At 4 weeks post-TBI, TBI relative to control for Ubiquitin and Ref2P is *p* < 0.05, according to two-way ANOVA, with Sidak’s multiple comparison correction. Representative blot images for head samples are included below graph. (**D–K**) TBI-treated fly heads show higher AMP gene (Drosomycin (**D,H**), Diptericin (**E,I**), Cecropin (**F,J**), and Drosocin (**G,K**)) expression at multiple timepoints, as compared to age-matched, uninjured controls. Six cDNA replicates of 20 fly heads each were prepared for each condition, at each timepoint: 2 h, 4 h, 8 h, 48 h, 2 weeks, and 4 weeks post-TBI; gene expression levels were calculated via standard curve for each gene, normalized to RpL1 gene expression levels, and then log-transformed. Two-way ANOVA with Sidak’s multiple comparisons test was performed to determine statistical significance between TBI and control at each timepoint. For the acute timepoints: (**D**) Drosomycin (Drs), (**E**) Diptericin (Dipt), and (**G**) Drosocin (Dro) are all significantly elevated (*p* < 0.0001) at 2, 4, and 8 h following TBI. (**F**) Cecropin (Cec) is significantly elevated (*p* < 0.0001) at 2 and 4 h post-TBI, as well as (*p* < 0.01) at 8 h post-TBI. Following the acute injury phase, the highest induction of AMP gene expression was observed at 48 h post-TBI, with the pairwise comparison between TBI and control for each AMP gene being statistically significant. At 2 weeks post-TBI, Dipt, Cec, and Dro, but not Drs, were significantly elevated. At 4 weeks post-TBI, no pairwise comparisons were significant, although Drs was trending with a *p* value of 0.0763.

We next examined two molecular markers associated with brain damage: protein aggregation and neuroinflammation. Both are classic hallmarks that characterize many different types of neurodegenerative diseases, as well as being understood as markers of general aging and TBI^3,7–9,11,13,22,52,53^. To examine protein aggregation, we measured levels of poly- and mono-ubiquitinated protein (P4D1) and Ref2P, which binds to poly-ubiquitinated proteins^54,55^ in insoluble protein extracts from the heads of injured and uninjured flies. Insoluble ubiquitinated proteins (IUP) and Ref2P are commonly used measures of protein aggregation in the *Drosophila* aging and neurodegeneration fields because proteins are targeted for degradation through poly-ubiquitination, and increased levels of IUP have been interpreted as pathological aggregates of proteins intended for degradation^56^. Insoluble ubiquitinated proteins and Ref2P have been shown to increase with age, as well as in fly models of human ND, both through Western blot analysis and immunostaining of brains, which often shows colocalization of Ref2P and ubiquitin puncta^54–57^. TBI-treated flies display a significant increase in both of these protein aggregation markers relative to age-matched, uninjured control flies at 4 weeks post-TBI, but not earlier (Fig. 2A,B). This is consistent with prior findings using a different TBI method in *Drosophila* that showed no significant differences in Ref2P and ubiquitinated proteins at 1 week post-TBI^41^. This long-term rise in protein aggregates appears to be head-specific, as we do not observe the same increase in body samples (Fig. 2C). This result suggests that the HIT assay has a specific and long-term effect on the head.

To examine neuroinflammation, we measured the expression of four antimicrobial peptide (AMP) genes in the heads of injured and uninjured flies: Diptericin (Dipt), Drosomycin (Drs), Drosocin (Dro), and Cecropin (Cec). In *Drosophila*, AMPs are highly expressed in response to infection or injury, as a result of activation of immune pathways (Toll and/or Imd)^58,59^. AMP gene expression is also known to increase with aging and play a role in modulating neurodegeneration in flies^60–63^. Consistent with published findings^37,41^, we observe a significant, acute increase in all AMP genes over several hours (2, 4, and 8 h) immediately following the TBI (Fig. 2D–G). In pairwise comparisons between TBI and controls for each timepoint, within each AMP, all four AMP transcripts are significantly elevated at 48 h following TBI (Fig. 2H–K), consistent with prior findings that some AMP genes remain elevated 1 week following a mild TBI^41^. All remain significantly elevated at 2 weeks post-TBI, except Drosomycin. None is significantly increased at 4 weeks, though the difference between TBI and control Drs transcript levels has a *p* value of 0.076 when corrected for multiple comparisons (Fig. 2H). This suggests a strong acute induction of the immune response that persists at high levels in the days and weeks following the injury, gradually returning close to baseline levels by the long-term timepoint but remaining modestly elevated—perhaps indicative of chronic neuroinflammation.

### TBI leads to starvation sensitivity and decreased lipid stores that persist through two weeks post-injury

Given that TBI in the fly leads to measurable, consistent molecular and physiological consequences similar to those seen in TBI and NDs, we next examined metabolic dysfunction. Metabolic disorders, such as diabetes and cachexia, are common co-morbidities of ND, but the timing of onset of metabolic disruption is not well characterized^19,22,27^. We set out to examine the timing of metabolic dysfunction following TBI in flies and gain a better understanding of its potential contribution to disease progression.

**Figure 3:**
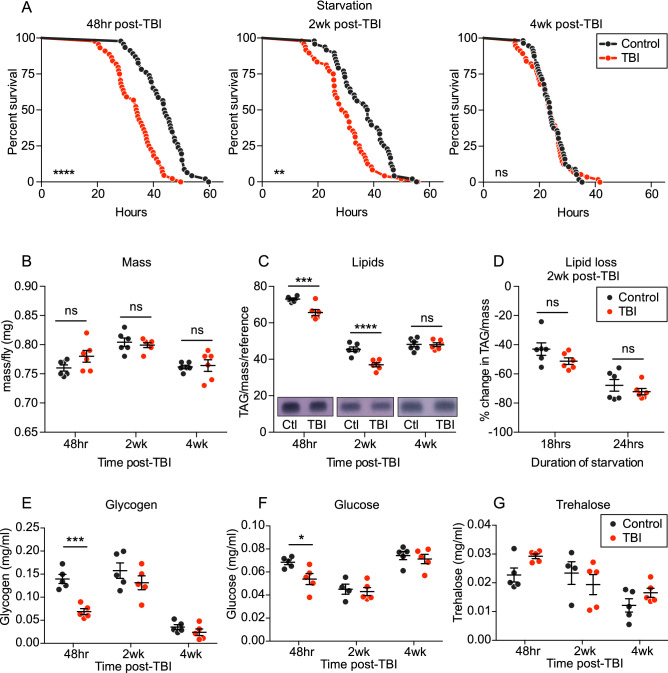
TBI induces metabolic dysfunction that disappears between 2 and 4 weeks following TBI. TBI-treated flies = red; age-matched, uninjured control flies = dark grey. (**A**) TBI-treated flies die faster under starvation than age-matched, uninjured controls at 48 h (*p* < 0.0001) and 2 weeks (*p* < 0.01) post-TBI, but not at 4 weeks post-TBI, according to both Log-rank and Gehan-Breslow-Wilcoxon survival comparison tests. Representative survival curves shown (44–50 flies per condition); at least three independent replicates were performed yielding similar results. (**B**) Mass is unchanged following TBI, according to a two-way ANOVA, with Sidak’s multiple comparison correction (six replicates of 20 flies each per group). (**C**) TBI-treated flies display lower lipid stores (triacylglycerides, TAG) at 48 h (*p* < 0.001) and 2 weeks (*p* < 0.0001) post-TBI, but not at 4 weeks post-TBI, according to two-way ANOVA, with Sidak’s multiple comparison correction. Lipids were extracted from TBI-treated and age-matched control flies (six replicates of 20 flies each per group) and measured in triplicate with thin-layer chromatography (TLC) and amido black stain (representative images shown), normalized to mass of individual sample and a reference sample of pure coconut oil, which was included on all TLC plates as a standard. (**D**) TBI-treated flies at 2 weeks post-TBI do not display an increased rate of lipid utilization during starvation. TBI-treated flies display similar reductions in lipid amount after 18 h and 24 h of starvation, as their age-matched, uninjured control flies, according to two-way ANOVA, with Sidak’s multiple comparison correction (six replicates of 20 flies each per group). TBI leads to short-term mobilization of carbohydrates, but returns to normal levels by 2 weeks. (**E**) Glycogen and (**F**) Glucose levels are significantly decreased (*p* < 0.001 and < 0.05, respectively) in TBI-treated flies relative to controls at 48 h post-TBI, but not at 2 or 4 weeks post-TBI. (**G**) Trehalose levels are not significantly different at any timepoint post-TBI. Significance determined by two-way ANOVAs, with Sidak’s multiple comparison tests, for each carbohydrate.

First, we measured starvation survival as a broad measure of metabolic function in the fly. Relative to controls, injured flies were sensitive to starvation at both short-term (48 h) and mid-term (2 weeks) timepoints post-TBI (Fig. 3A). This difference between controls and injured flies disappeared by the long-term timepoint of 4 weeks post-TBI. To investigate why injured flies starved faster at short- and mid- term timepoints, we asked whether they had a defect in energy storage or energy usage. While injured flies do not display any significant differences in overall mass (Fig. 3B), they do exhibit decreased triacylglycerides, or TAGs (the major component of lipid energy stores^64–66^), at short- and mid-term but not long-term post-TBI timepoints, mirroring the pattern observed in starvation sensitivity (Fig. 3C). We also measured levels of a short-term energy molecule: carbohydrates (glycogen, glucose, and trehalose), and discovered a short-term disruption in carbohydrates at 48 h following the injury, leading to a decrease in glycogen and glucose (Fig. 3E–G). However, this difference disappeared by 2 weeks post-TBI, suggesting that the mid-term starvation sensitivity may be more dependent on the lipid storage defect. We also investigated whether injured flies exhibit a mid-term change in energy usage by measuring the reduction in their lipid levels during starvation. Both injured and control flies displayed a similar ~ 50% reduction in TAG levels after 18 h of starvation, and ~ 70% reduction after 24 h (Fig. 3D), indicating rate of lipid utilization is not significantly altered by TBI. Taken together, these results suggest that TBI causes changes in overall metabolism that persist for weeks after injury but eventually resolve or become indistinguishable from age-related changes and that these metabolic changes appear to be due to a defect in lipid storage rather than usage.

### Dietary restriction (DR) treatment ameliorates some TBI-induced phenotypes

Because this TBI model induces many phenotypes characteristic of classical aging (locomotor decline, protein aggregation, inflammation, metabolic defects), we investigated the impact of a metabolic intervention called dietary restriction (DR) on TBI outcomes. DR treatment is known to ameliorate many classical aging phenotypes^67,68^, and is commonly applied in *Drosophila* aging research via a reduction of protein content (yeast extract) in *Drosophila* media. We therefore implemented a DR treatment paradigm in which flies were reared on a commercial food, but then starting within 24–48 h of eclosion, were maintained on either DR food containing 1% yeast extract (1% YE), or an otherwise identical, non-DR food containing 6% yeast extract (6% YE).

**Figure 4:**
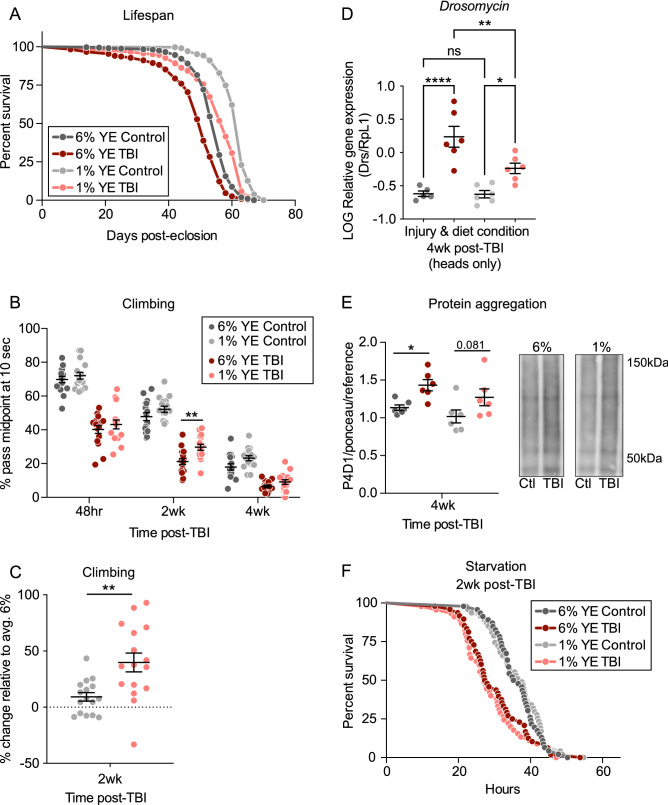
Dietary restriction treatment partially ameliorates some TBI-induced phenotypes. DR-fed (1% YE), TBI-treated flies are shown in pink, and 1% YE control flies are shown in light grey. Standard-fed (6% YE), TBI-treated flies are shown in maroon, and 6% YE control flies are shown in grey. (**A**) 1% YE TBI-treated flies have significantly increased lifespan relative to both 6% YE TBI and control flies. TBI still leads to decreased lifespan in both 1% YE and 6% YE flies. All comparisons *p* < 0.0001, according to both Log-rank and Gehan-Breslow-Wilcoxon Survival comparison tests. One of three independent lifespan experiments displayed (sample size of 205–319 flies per group). (**B**) 1% YE TBI-treated flies exhibit significantly increased climbing ability at 2 weeks post-TBI, relative to 6% YE TBI flies (*p* < 0.01), according to two-way ANOVA, with Sidak’s multiple comparison correction (at least 16 groups of 20–25 flies each). TBI still induces a significant decrease in climbing ability at all timepoints, in both 1% YE and 6% YE groups (significance not indicated on graph). (**C**) The percent change in climbing ability of 1% YE flies (TBI in pink and control in light grey), relative to the average climbing ability of 6% YE flies of the same injury condition was calculated. At 2 weeks, the percent change in climbing ability of 1% YE relative to 6% YE flies is significantly increased (~ 40%) in TBI-treated flies but not control flies. DR-related change in climbing ability is also significantly increased in TBI-treated, but not control flies at 4 weeks post-TBI (data not shown). Both are determined by a two-way ANOVA, with Tukey’s multiple comparison test (*p* < 0.05). (**D**) Both 1% YE (*p* < 0.05) and 6% YE (*p* < 0.0001) TBI-treated fly heads show higher Drosomycin gene expression at 4 weeks post-TBI, when compared to age-matched, uninjured controls in each diet condition (six replicates of 20 fly heads each per condition). 1% YE TBI-treated flies display significantly decreased Drs gene expression relative to 6% YE TBI-treated flies (*p* < 0.01), but 1% YE controls do not exhibit significantly different Drs gene expression relative to 6% YE controls. Gene expression levels were calculated via standard curve, normalized to RpL1 gene expression levels, and then log-transformed. *p* values determined from one-way ANOVA pairwise comparisons between selected injury and diet conditions, with Sidak’s multiple comparisons test. (**E**) Mono- and poly-ubiquitinated protein between ~ 40 and 150 kDa, measured by P4D1 antibody, levels in head samples (from insoluble protein extracts of 6 replicates of 50 heads each) are significantly elevated (*p* < 0.05) in 6% YE TBI-treated flies, and trending (*p* = 0.081) elevated in 1% YE TBI-treated flies. TBI and control samples within each diet condition were run on the same western blot, normalized to total protein between 37 and ~ 200 kDa via Ponceau stain, and then normalized to a standard reference protein sample of mixed young and old flies, which was included on each western blot. Significance determined by two-way ANOVA, with Sidak’s multiple comparison test. Representative blot images shown. (**F**) 1% YE flies (TBI or control) do not exhibit a change in starvation sensitivity, relative to their 6% YE counterparts, at 2 weeks post-TBI. TBI-treated flies (both 1% YE and 6% YE) still exhibit a significant decrease in survival under starvation (sample size of 43–48 flies per condition).

Dietary restriction (1% YE) increased the lifespan (excluding acute mortality) of injured flies, relative to injured flies fed standard food (6% YE) (Fig. 4A). A similar DR-induced lifespan increase was observed with uninjured controls (Fig. 4A). Interestingly, 1% YE TBI flies also displayed a significantly increased lifespan relative to 6% YE uninjured, control flies. Additionally, DR treatment specifically improved the climbing ability of injured flies at 2 weeks post-TBI, resulting in a ~ 40% increase in climbing ability of TBI flies, but not control flies (Fig. 4B,C). However, the DR treatment (1% YE) did not eliminate the TBI-induced climbing defect relative to uninjured control flies (either 1% YE or 6% YE) (Fig. 4B). At 4 weeks post-TBI, there was not a significant difference in climbing pass rate between 1% YE TBI and 6% YE TBI.

We also observed a significant decrease in Drs gene expression in heads of 1% YE TBI flies relative to 6% YE TBI flies at 4 weeks post-TBI (Fig. 4D). DR treatment specifically impacted Drs gene expression levels in *injured* flies, as there was no significant difference between uninjured control 1% YE and 6% YE conditions. This DR-induced reduction in expression was not observed for the three other AMP genes tested (Fig. S1A). For each gene, TBI significantly increased the expression relative to uninjured controls fed the same diet, further supporting the finding of modest, long-term neuroinflammation post-TBI.

At 4 weeks post-TBI, we did not observe a meaningful change in the TBI-induced increase in protein aggregation in 1% YE flies (Fig. 4E); a significant, or nearly significant, increase was observed in both 1% and 6% YE TBI, relative to controls. Though DR, a dietary intervention, ameliorated a variety of classic neurodegenerative and aging phenotypes in injured flies, it did not impact TBI-induced starvation sensitivity (Figs. 4F, S1B). Taken together, this suggests that *some* DR-influenced pathways lead to TBI-induced phenotypes, but other pathways independent of DR may also be disrupted.

### TBI-induced phenotypes exhibit a variety of patterns and intensities over time

**Figure 5:**
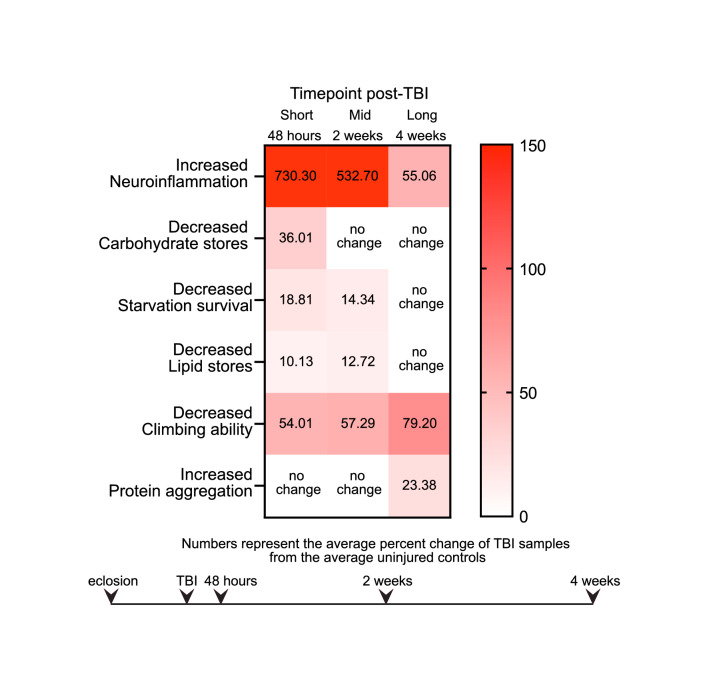
TBI induces molecular and physiological changes with different temporal patterns. Average percent change values of TBI-treated flies relative to the average uninjured control value at each timepoint post-TBI were calculated for samples that displayed a statistically significant difference. Increasing shades of red represent greater magnitudes of TBI-induced change in the phenotype (see left-hand description for increase or decrease relative to uninjured controls). Scaled timeline of when the injury is delivered and when timepoints are measured, starting with day of eclosion is displayed.

To compare the kinetics and relative magnitudes of TBI-induced phenotypes, we calculated the average percent change of the TBI samples, compared to the average of the control samples, for each phenotype and timepoint in which there was a statistically significant difference between groups (Fig. 5). The largest magnitude changes were observed in increased AMP gene expression (~ 500–700% at 48 h and 2 weeks post-TBI, averaged across all 4 AMP genes measured), though this waned by 4 weeks post-TBI. Relatively modest magnitude changes (~ 10–20%) occurred in the metabolic phenotypes of decreased lipid stores and starvation and this disappeared by 4 weeks post-TBI. In contrast to these early onset immune and metabolic changes, a ~ 23% increase in protein aggregation was only observed at 4 weeks post-TBI. The timing of onset of molecular and physiological changes may suggest causality, with earlier changes contributing to later occurring ones. Thus, this provides a framework to begin manipulating early phenotypes, such as immune and metabolic pathways, and measure if they alter later outcomes such as protein aggregation.

## Discussion

We characterized short-, mid-, and long-term outcomes of a traumatic brain injury *Drosophila* model^37^, focusing on those outcomes that phenocopy symptoms common to many neurodegenerative diseases. Our aim was to identify early physiological and molecular changes that may contribute to the progression of long-term neurodegenerative decline following a brain injury. TBI-induced phenotypes exhibited a wide range of onsets and patterns, with some diminishing or resolving with age and time following the injury and others increasing or only appearing weeks later. AMP gene expression was the only phenotype measured during the acute phase post-TBI and all four AMP genes measured were highly elevated in TBI-treated flies. They remained strongly elevated short-term and persisted as modestly elevated mid-term and returned close to baseline long-term. The decrease in carbohydrates, glycogen and glucose, observed at 48 h post-TBI, was no longer different from controls by the mid-term timepoint, whereas the broader metabolic disruption characterized by starvation sensitivity and decreased lipids was present at both short- and mid-term timepoints and only disappeared at a long-term (4 weeks) timepoint following the injury. The TBI-induced locomotor decline was similarly significant at short- and mid-term phases but increased in magnitude by the long-term timepoint. Protein aggregation levels were not significantly increased at short- and mid-term timepoints but were elevated exclusively at 4 weeks post-TBI. Recent prior studies using a variety of methods to inflict TBI in *Drosophila* also demonstrated some of the TBI-induced phenotypes we observed, including AMP gene expression, climbing deficits, and intermediate-term (1–2 weeks) protein aggregation, though the phenotypes are not often measured at multiple timepoints over several weeks^37,41,46,48–51,69^. To our knowledge, the characterization of TBI-induced phenotypes at 4 weeks post-TBI, starvation sensitivity and decreased lipid stores, as well as DR’s effect on TBI-induced phenotypes, have not been previously published.

An observation that highlights the importance of studying the timing of different phenotypes is that the TBI-induced starvation sensitivity and decreased lipid stores have the same pattern of timing (differences present at 48 h and 2 weeks post-TBI, but not at 4 weeks post-TBI). This suggests that these phenotypes are related, which is consistent with the hypothesis that the TBI-treated flies’ decreased lipid stores lead to decreased survival time during starvation, as has been observed in prior studies of genetic disruption of lipid homeostasis that lead to starvation sensitivity or resistance^70,71^. The acute and short-term response to any severe injury is metabolically intensive^72^, so the short-term decrease in lipid and carbohydrate levels observed at 48 h post-TBI is consistent with an immediate mobilization of energy stores to repair the injury. However, the persistence of decreased lipids, and the accompanying starvation sensitivity, through at least 2 weeks post-TBI, suggests a significant TBI-associated disruption in metabolic regulation, beyond an immediate injury repair response. The apparent resolution of the TBI-induced starvation sensitivity at 4 weeks results from the increased starvation sensitivity of uninjured control flies as they age, while the TBI flies’ starvation sensitivity remains similar to what is observed at 2 weeks. These results suggest the possibility of an “accelerated aging” phenotype resulting from the TBI.

In human ND clinical research, there has been mounting evidence of an association between metabolic dysfunction and neurodegeneration, though the causality of this association is not always clear^19^. Mammalian and fly models of NDs have shed light on molecular pathways that may underlie metabolic dysfunction’s role in neurodegeneration^29,73–75^. Interestingly, in a *Drosophila* model of Huntington’s Disease, increased lipids were associated with early stages of disease (first several days), while diseased flies started to exhibit decreased lipids by 1.5 weeks of age^76^. Since we observed that TBI caused a reduction in stored lipids, a reduction that disappeared by 4 weeks post-TBI, it is possible that this represents a protective mechanism, rather than a dysregulation that drives pathology. Further studies, including genetic manipulation of lipid storage pathways, will clarify the role that lipid dysregulation plays in TBI-induced neurodegeneration.

Previous studies have demonstrated the beneficial impact of dietary restriction (DR) on features of aging, including lifespan and locomotor decline^77–80^, and neurodegenerative disease models specifically^74,81^. Interestingly, while we observe the expected DR-induced lifespan increase in control flies, we do not observe an increase in locomotor performance in control flies due to DR, or DR-related starvation resistance in either TBI or control flies, which has been previously reported in female flies^82^. This could be due to the use of male flies, instead of female flies, which exhibit a greater lifespan response to DR^83^ and greater starvation resistance than males under normal dietary conditions^84^. For locomotor performance, there is a great deal of technical variation in the execution and analysis of climbing assays that might alter our ability to observe a more subtle DR-induced increase in climbing performance in the control flies.

Furthermore, some studies of mammalian TBI models have demonstrated that dietary restriction can ameliorate tissue damage and cognitive decline following the injury^85,86^. We found that DR treatment did improve multiple TBI-induced phenotypes (lifespan, climbing, and Drs expression), while never completely eliminating the difference between TBI and control flies both fed DR. Surprisingly, DR treatment led to these benefits without altering the metabolic dysfunction induced by TBI, as measured by starvation sensitivity. We had hypothesized that if the DR treatment had an impact on the neurodegenerative phenotypes, it would do so by modifying the altered metabolic regulation induced by TBI. However, it is possible that the pathways that lead to the metabolic disruption following TBI are distinct from the ones that DR is acting on to improve lifespan, locomotor function, and *Drosomycin* expression. Perhaps the DR treatment is not enacting its beneficial effect through predominantly *metabolic* pathways (e.g., TOR signaling) that would influence starvation sensitivity, but through other pathways that DR is known to influence, such as oxidative stress response and immune pathways^87–90^. The DR-induced reduction in *Drs,* but not *Dipt*, *Cec*, or *Dro*, gene expression might suggest a greater impact of DR on Toll signaling than Imd^91,92^, specifically in TBI flies, though further investigation is needed. Alternatively, because TBI leads to a “leaner” phenotype of lower energy stores and sensitivity of starvation, perhaps the pathways that DR would act through are already maximally perturbed. Given the modest DR-induced effects observed, it is worth noting the possibility that the assays used were not sensitive enough to detect a small change due to DR. For example, there is no apparent effect of DR on 4 week post-TBI climbing, only at 2 weeks post-TBI, but the dynamic range of climbing ability at that age is significantly reduced in control flies, making it difficult to detect significant small changes in climbing behavior.

Additionally, the DR treatment does not seem to robustly impact the phenotype that takes the longest to appear: protein aggregation. While detection sensitivity could prevent us from observing a modest reduction in protein aggregation due to DR, it is also possible that the pathways that lead to the TBI-induced protein aggregation are independent of those that DR influences to ameliorate climbing and lifespan. Perhaps protein aggregation is linked to the pathway underlying the starvation sensitivity, which similarly does not appear to be altered by DR treatment. Ultimately, it is likely that many different pathways are altered in the secondary injury responses triggered by TBI and that these alterations persist over different timescales, leading to different long-term outcomes related to neurodegeneration. One strength of using *Drosophila* is the ability to use a wealth of genetic tools to perturb different pathways and observe how they modify these TBI-induced outcomes.

Using an injury-based model as a broader way of studying neurodegeneration offers unique advantages, such as the ability to observe the timing of early TBI-induced changes while avoiding developmental effects of some mutant models. It may allow us to identify important common pathways that underlie diverse neurodegenerative diseases and illuminate links between TBI and neurodegeneration. Examining early stages following the injury to identify mechanisms of pathogenesis will allow us to identify potential targets for intervention that may be preventative and reduce disease progression as an individual ages.

## Methods

### Fly lines and husbandry

Male flies of the wild-type strain, Canton-S, were used for all experiments. Males were collected 0–2 days after eclosion and allowed to mate for 24–48 h before being separated from females. Flies were reared and maintained on standard cornmeal-agar media (Archon Scientific® Glucose recipe, http://archonscientific.com/) in a 25 °C, humidity controlled (55–65%), 12:12 light:dark incubator. Flies used for cDNA samples in Fig. 2 QRTPCR were reared and maintained on molasses media (due to the timing of when these samples were generated). During aging maintenance, flies were transferred to fresh vials every 2–3 days. For dietary restriction (DR) experiments, flies were reared on Archon Glucose media, and then placed on 1% or 6% YE media 1–3 days after eclosion and maintained for the duration of experiments. Media for DR experiments contained 5% cornmeal, 4% dextrose, 2% sucrose, 1% agar, 0.16% Tegosept, 1% propanoic acid, and either 6% (non-DR) or 1% (DR) yeast extract.

### Traumatic brain injury (TBI) paradigm

TBIs were inflicted using a High Impact Trauma (HIT) device and protocol minimally modified from Katzenberger et al.^37^. The HIT device consists of a spring with one end fixed to a wooden board and the other end free, such that a vial of flies to be injured can be attached. The only changes are transferring of the flies into the HIT device without anesthesia (flies are separated into vials of appropriate sex and number at least 24 h prior and then simply passed into the HIT vial with a funnel) and use of a small rod to ensure a consistent angle of deflection. To deliver the TBI treatment, approximately 40–45, 5–7 day old mated male flies were placed in an empty vial and attached to the spring. The spring was pulled back to 90° and released, allowing the vial to impact a plastic pad below, delivering an injury to the flies inside. Flies underwent 4 strikes spaced 5 min apart. Age-matched control flies were transferred to identical empty vials for the same amount of time as injured flies, but were not given the TBI treatment. All flies were transferred to fresh food vials placed on their sides to recover in a 12:12 light:dark, humidity-controlled, 25 °C incubator for 48 h. Acute mortality, the percentage of flies that died within 48 h, was measured and dead flies were excluded from all subsequent experiments.

### Lifespan assay

For lifespan analysis, at least 10 vials with approximately 20–30 flies per vial were passed to fresh media every 2–3 days and death was recorded. Experiments began 48 h after flies underwent HIT protocol, therefore acute death from TBI was excluded. Three independent repeats were conducted at different dates for each experiment.

### Climbing assay

To measure climbing ability, 20–30 flies were transferred without anesthesia to an empty narrow polystyrene vial (25 mm diameter) and topped with a second identical vial to form an enclosed climbing vial approximately 20 cm high. After allowing flies to acclimatize to the climbing vial laid on its side for 10 min, up to 8 vials were loaded side by side into an enclosed clear plastic apparatus fitted such that the vials were held tightly. Humidity was controlled within the apparatus with a moistened paper towel and monitored with humidity probe. All assays were performed between 60 and 80% humidity and at a room temperature of 20–23 °C. To start the assay, the apparatus was vigorously tapped down so that all flies fell to the bottom of their vials. Flies were then allowed to climb for at least 1 min. The apparatus was tapped down 4 more times for a total of 5 trials. The entire process was filmed for later analysis. Flies were then cold- or CO_2_-anesthetized briefly after the assay to allow the precise number in each vial to be counted. Flies were recovered onto fresh food for later experiments. The same groups of flies were repeat tested at each timepoint: 48 h, 2 weeks, and 4 weeks post-TBI.

Videos of climbing assays were manually analyzed. The time at which the apparatus was tapped down was recorded, the number of flies past the midpoint in each vial after 10 s was counted and then converted into the percentage of the total number of flies. The percentages past midpoint of the five trials were averaged for a single data point.

### Western blotting and antibodies

Injured flies and age-matched control flies were collected at various timepoints post-TBI and frozen at -80 °C. Protein was extracted from 50 heads or 20 bodies, per biological replicate sample. Flies were flash frozen in liquid nitrogen and vortexed for 30 s to separate heads from bodies. Tissue was initially homogenized with electric pestle grinder in a 1% Triton-X100 Homogenization Buffer (THB), containing protease & phosphatase inhibitors, centrifuged at 4 °C for 10 min, after which the soluble protein fraction was removed and combined 1:1 with a 2 × LDS buffer (containing protease & phosphatase inhibitors, and 80 mM DTT). The remaining insoluble protein pellet was washed by homogenization with 1% THB, 4 °C centrifugation, discarding the supernatant. The remaining insoluble pellet was homogenized with 2 × LDS buffer, heated at 100 °C for 5 min, then centrifuged for 10 min at room temperature.

Protein samples were run on a 3–8% Tris-acetate gel, then transferred to PVDF membrane using Bio-Rad Turboblot semidry transfer apparatus. Membranes were stained with a Ponceau solution and imaged on an AI600 imager on colorimetric setting to measure total protein. Membranes were then blocked in 3% BSA for an hour at room temperature before being incubated in primary antibody for 48–72 h at 4 °C. Blots were incubated with the appropriate secondary antibody solution for 2 h at room temperature, then ECL solution was applied prior to chemiluminescence imaging with an AI600 imager. Images were quantified and background subtracted using FIJI software, and divided by total protein in each sample from Ponceau measurement of ~ 37 to 200 kDa blot. Each sample/ponceau value was then divided by the sample/ponceau value of the same reference protein sample (from wild-type mixed age flies), included in all blots for a particular dataset. The following primary antibodies were diluted in 3%BSA: anti-P4D1, measuring ubiquitin, mono-, and poly-ubiquitinated proteins (CST #3936, mouse, at 1:2000), anti-Ref2p (Abcam ab178440, rabbit, at 1:500). The P4D1 antibody detects free ubiquitin (~ 9 kDa), mono-, and poly- ubiquitinated proteins. We quantify a protein size range on the Western blot such that excludes free ubiquitin and captures mono- and poly-ubiquitinated proteins. Secondary antibodies used were either anti-mouse IgG-HRP (CST #7076) or anti-rabbit IgG-HRP (CST #7074) at a concentration of 1:2000 in 3% BSA.

### Quantitative real-time PCR

Injured flies and age-matched control flies were collected at various timepoints post-injury and frozen at − 80 °C. RNA was extracted from 20 fly heads per biological replicate using Trizol (Invitrogen), according to the manufacturer’s protocol. DNasel (Invitrogen) was used to treat each sample, then heat inactivated. RNA concentration was determined and normalized to lowest sample’s concentration prior to cDNA synthesis. cDNA was synthesized using Revertaid First Strand cDNA Synthesis Kit (Thermo Scientific). QRT-PCR was performed with a Bio-Rad CFX Connect Real-Time PCR machine, using Express Sybr GreenER qPCR SuperMix (Invitrogen). Primer efficiency and relative quantification of transcript level was determined by creating a standard curve for each primer set using serial dilutions of cDNA. Transcripts were normalized to ribosomal protein L1 (RpL1) transcript levels. ROUT (Q = 1%) method of outlier detection was used to eliminate 1 outlier sample from each of the AMP 4-week post-TBI datasets from Fig. 4 and S1 (see supplemental raw data Excel file). AMP/RpL1 gene expression levels were then log (base 10) transformed prior to applying the appropriate statistical tests (one- or two-way ANOVAs).

Primer sequences used were as follows:

- Diptericin (Dipt): accgcagtacccactcaatc; cccaagtgctgtccatatcc
- Drosocin (Dro): ccatcgaggatcacctgac; ctttaggcgggcagaatg
- Cecropin (Cec): tcttcgttttcgtcgctctc; cttgttgagcgattcccagt
- Drosomycin (Drs): gtacttgttcgccctcttcg; cttgcacacacgacgacag
- Ribosomal Protein L1 (RpL1): tccaccttgaagaagggcta; ttgcggatctcctcagactt.

### Starvation assay

Starvation assays were performed using Trikinetics, Inc. *Drosophila* Activity Monitors (DAMs). Individual flies were placed in 5 mm tubes containing 1% agarose in DAMs in a 12:12 light:dark, humidity-controlled, 25 °C incubator for 3–5 days. Activity data was summed into 10-min bins and time of death was determined as the time immediately after last recorded movement. For each starvation experiment, 32–48 flies were used per group, and at least 3 independent repeats of each experiment were performed on different dates.

For dietary restriction (DR) experiments described in Figs. 4 and S1, flies were placed on 1% or 6% YE food 1–3 days after eclosion and maintained throughout until the experiment. So for 48 h post-TBI flies, they were on DR or standard food for 5–7 days prior to the assay; for 2 weeks post-TBI, the flies were on food treatment for 19–21 days prior to testing; for 4 weeks post-TBI, the flies were on food treatment for 33–35 days prior to the starvation assay.

### Mass and triglyceride measurement

Injured flies and age-matched control flies were collected at various timepoints post-injury and placed in groups of 20 flies per biological replicate sample to weigh. Samples were then homogenized in a 2:1 chloroform:ethanol solution to solubilize triglycerides and some diglycerides, then centrifuged for 10 min at 4 °C. Samples were spotted on a TLC plate (Sigma Aldrich Silica gel on TLC-PET foils 99577-25EA) and separated with a solvent solution of 140 ml heptane, 60 ml diethyl ether, and 2 ml acetic acid. Samples were run in triplicate. A reference sample of 0.78125 mg/ml pure coconut oil in a 2:1 chloroform:ethanol solution was included on each plate and used to normalize sample values across plates. Plates were stained using 0.2% amido black (NAPHTHOL BLUE BLACK, Sigma Product N-3393-100G) in 1 M NaCl and imaged with an AI600 imager on colorimetric setting. Images were quantified in triplicate using FIJI. All values were divided by the mass of the sample measured prior to lipid extraction, and the coconut oil reference sample for the plate.

### Carbohydrate analysis

To prepare carbohydrate samples, injured and uninjured control flies were collected at 48 h, 2 weeks, and 4 weeks timepoints and groups of 10 flies per replicate (6 replicates per condition/timepoint) were washed several times with PBS, then homogenized in 200ul PBS. Samples were centrifuged for 1 min at 4 °C, and the supernatant removed and heated for 10 min at 70 °C. Samples were then centrifuged again for 3 min at 4 °C, and the supernatant was removed and diluted 1:4 with PBS. Glycogen was measured via glucose detection with Glucose (HK) Assay kit (Sigma GAHK20) after 1-h, 37 °C amyloglucosidase digestion. Trehalose was measured via glucose detection with Glucose (HK) Assay kit (Sigma GAHK20) after overnight, 37 °C trehalase digestion. Glucose values are from the non-digested samples from trehalose detection. Glucose (GO) Assay kit (Sigma GAGO20) was used to measure 2 week post-TBI trehalose and glucose samples. Absorbance was measured via a plate reader at 25 °C. Sample concentrations were determined via a standard curve with 1:2 serial dilutions of known concentrations of glucose.

### Statistical analysis

Statistical analysis was done using GraphPad Prism 6 software, or GraphPad Prism 9 software for QPCR data. Two-way ANOVAs were used for comparisons between two or more groups, with more than one measurement, and a Tukey or Sidak test for multiple comparisons (across multiple timepoints or measurements) was applied when appropriate. One-way ANOVAs were used for comparisons between two or more groups, with a single measurement, and a Sidak test for multiple comparisons was applied.

Survival curves (lifespan and starvation) were plotted as Kaplan–Meier graphs and log-rank analysis was performed to test statistical significance. Median survival times were used to calculate percentage differences in survival. All experiments were conducted with at least 3 independent trials that yielded statistically similar results. The graphs and *p* values displayed in figures are from an individual representative trial. Data from all trials can be found in supplemental Excel file “Fig1Fig4_Lifespandata.xlsx”.

Specific details of significance, and multiple comparisons made are reported in legend for each figure. In general, *p* < 0.05 (*), *p* < 0.01 (**), *p* < 0.001 (***), *p* < 0.0001 (****).

## Supplementary Information

Supplementary Figure S1.

Supplementary Information 2.

Supplementary Information 3.

Supplementary Information 4.

Supplementary Information 5.

Supplementary Information 6.

Supplementary Information 7.

Supplementary Information 8.

Supplementary Table S1.

## Data Availability

All datasets generated and/or analyzed during this study are provided in supplementary Excel data files.
