# Supplementary material for: Dietary restriction ameliorates TBI-induced phenotypes in Drosophila melanogaster
Source: Sci Rep. 2022 Jun 9;12:9523. doi: 10.1038/s41598-022-13128-x (PMC9184478; doi:10.1038/s41598-022-13128-x)
Supplement: Supplementary file 1 — Supplementary Figure S1. [file 41598_2022_13128_MOESM1_ESM.pdf]

Delventhal et al 2021 Figure S1

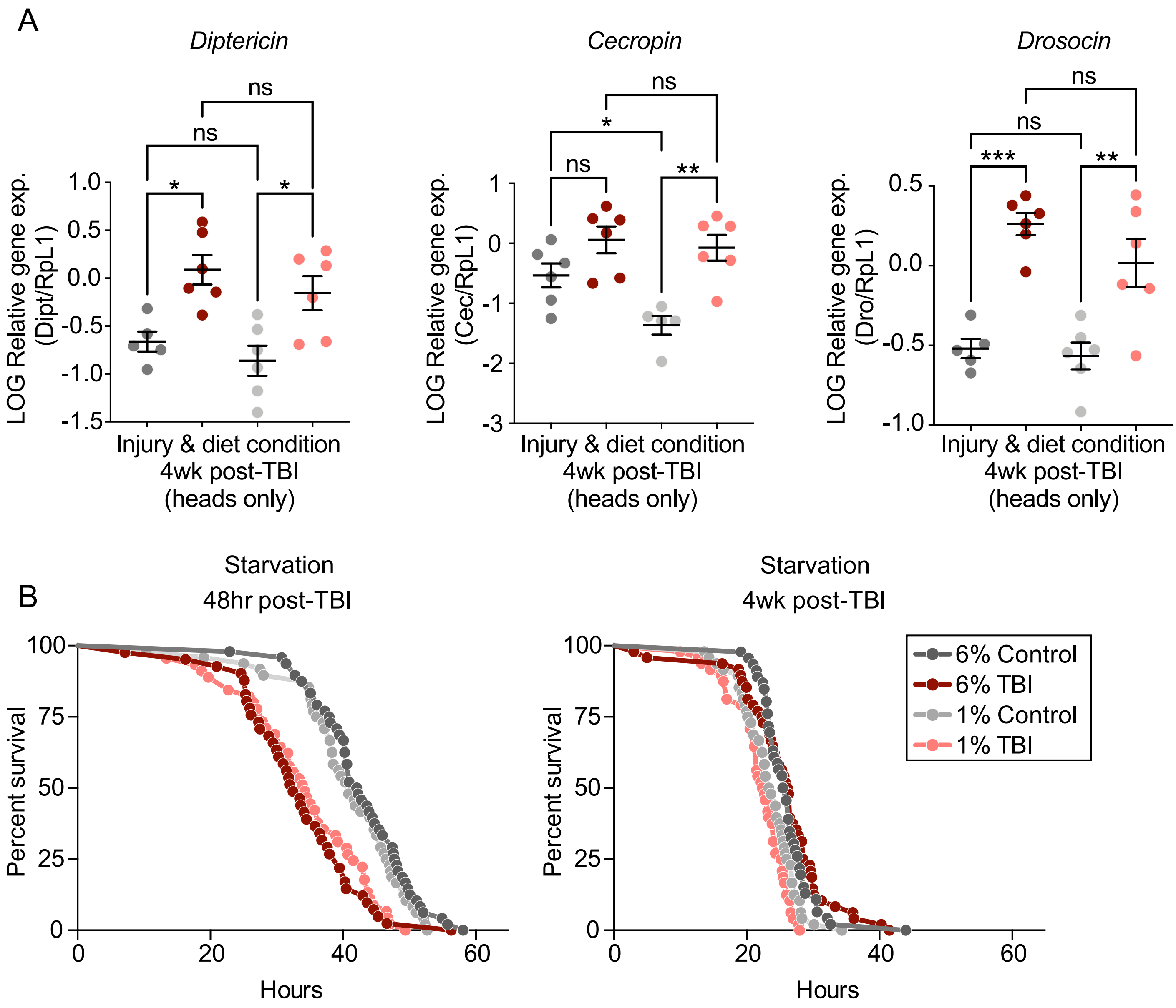

Figure S1. DR treatment does not impact most AMP gene expression or starvation sensitivity at 48 hours or 4 weeks post-TBI. DR fed (1% YE) TBI-treated flies are shown in pink, and 1% YE control flies are shown in light grey. Standard diet fed (6% YE) TBI-treated flies are shown in maroon, and 6% YE control flies are shown in grey. **(A)** Both 1% YE and 6% YE TBI-treated fly heads show higher Diptericin and Drosocin gene expression at 4 weeks post-TBI, when compared to age-matched, uninjured controls in each diet condition. 1% YE TBI-treated fly heads, but not 6% YE TBI-treated, show higher Cecropin expression relative to uninjured controls. None of these three AMPs (Dipt, Cec, Dro) show significant differences in expression between 1% YE TBI-treated flies and 6% YE TBI-treated flies. Gene expression levels were calculated via standard curve, normalized to RpL1 gene expression levels, and then log-transformed. Significance determined by one-way ANOVA pairwise comparisons between selected injury and diet conditions, with Sidak's multiple comparisons test. **(B)** 1% YE flies (TBI or control) do not exhibit a change in starvation sensitivity, relative to their 6% YE counterparts, at 48 hours or 4 weeks post-TBI. TBI-treated flies (both 1% YE and 6% YE) still exhibit a significant decrease in survival under starvation at 48 hours post-TBI that disappears by 4 weeks post-TBI.
