# Supplementary material for: Dietary restriction ameliorates TBI-induced phenotypes in Drosophila melanogaster
Source: Sci Rep. 2022 Jun 9;12:9523. doi: 10.1038/s41598-022-13128-x (PMC9184478; doi:10.1038/s41598-022-13128-x)
Supplement: Supplementary file 2 — Supplementary Information 2. [file 41598_2022_13128_MOESM2_ESM.docx]

**Figure 2A – Ubi heads**

48hr

ladder


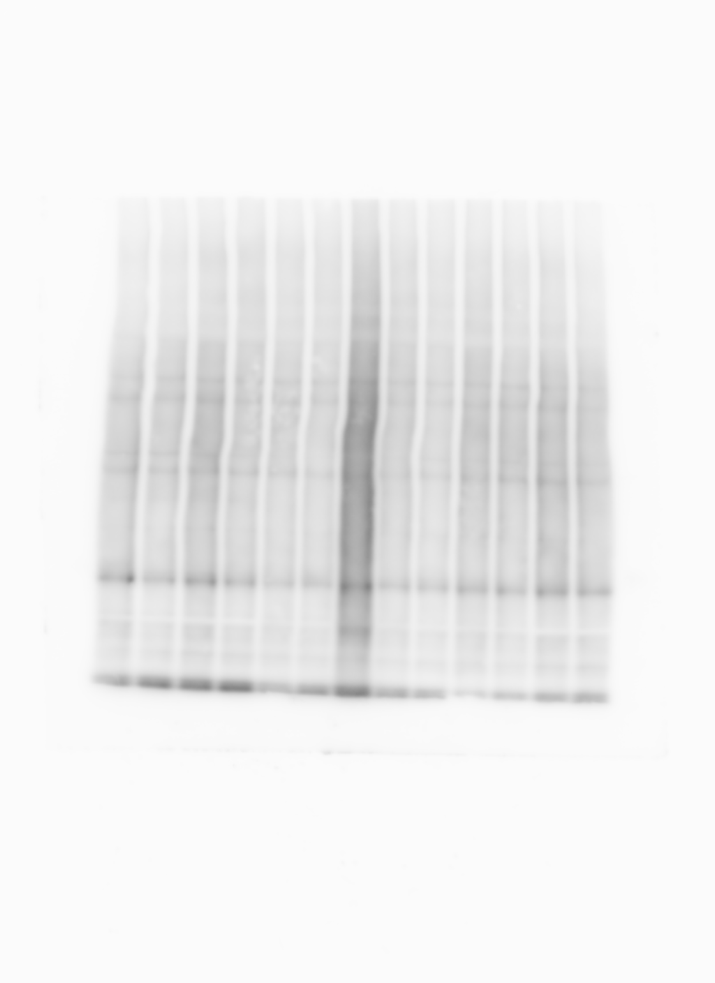


CTL H5

TBI H6

CTL H6

ladder

REF

TBI H1

TBI H2

TBI H3

TBI H4

TBI H5

CTL H1

CTL H2

CTL H3

CTL H4

2wk

CTL H6

TBI H6

CTL H5

TBI H4

TBI H5

CTL H4

CTL H3

TBI H3

CTL H2

TBI H1

TBI H2

CTL H1

REF

ladder

ladder


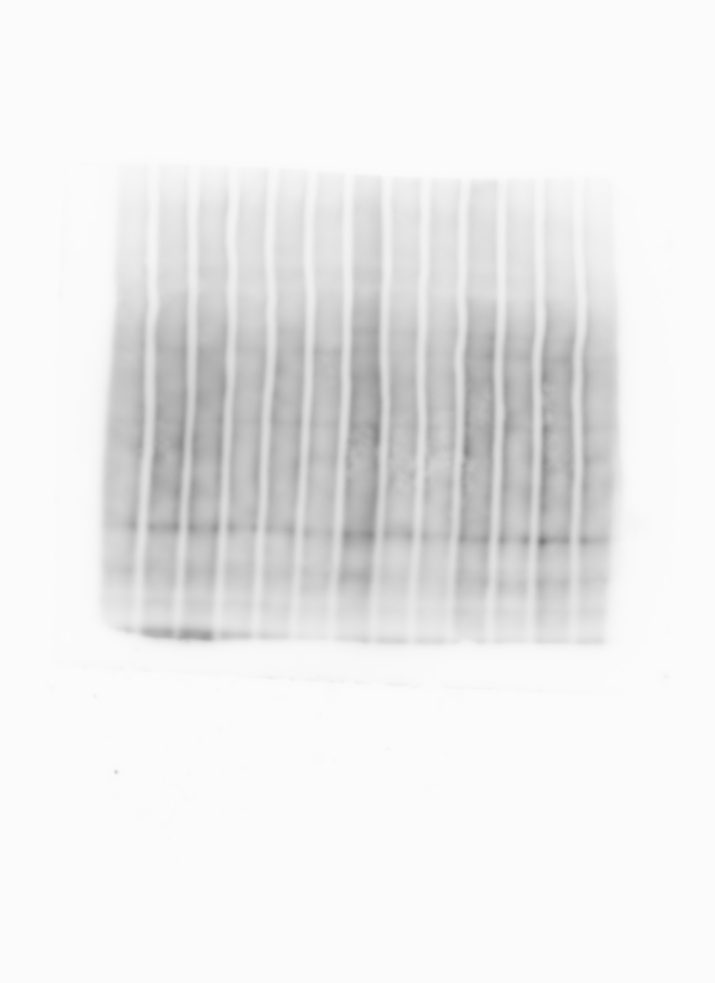


4wk

TBI H2

CTL H2

TBI H3

CTL H3

TBI H4

CTL H4

CTL H5

TBI H5

CTL H1

TBI H1

ladder

ladder

REF

CTL H6

TBI H6


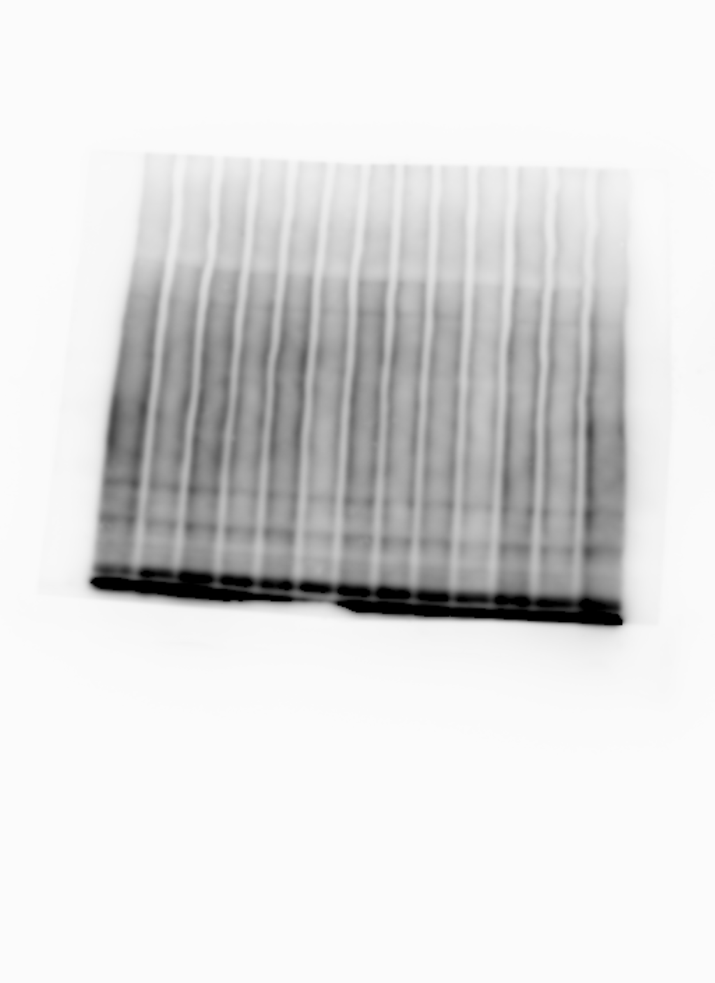


**Figure 2B – Ref2P heads**

48hr


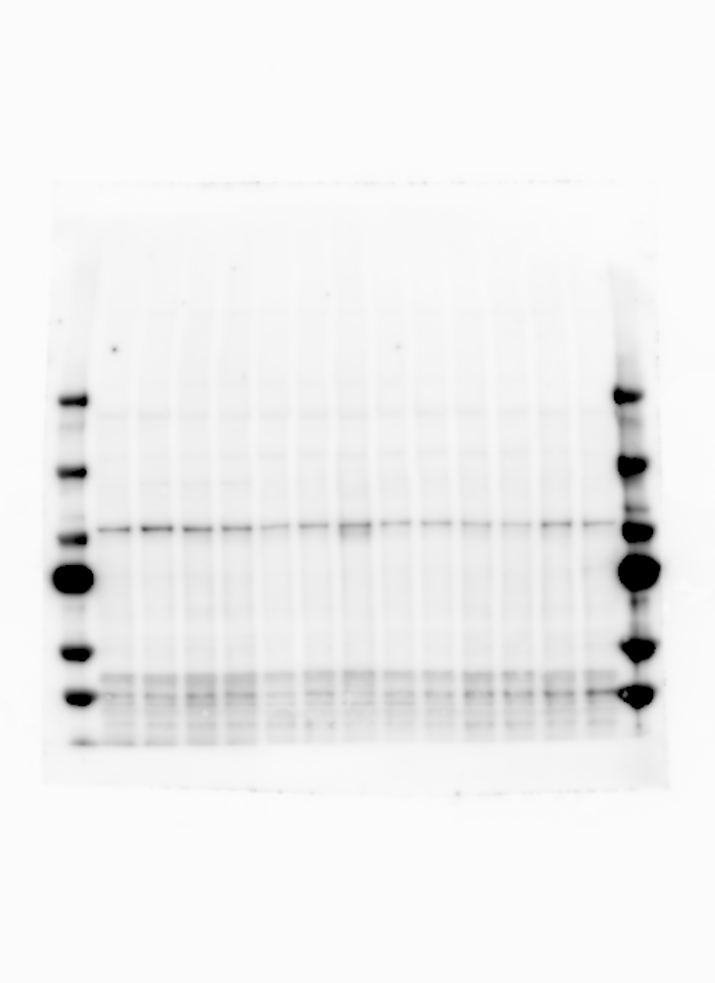


ladder

ladder

REF

TBI H6

CTL H6

CTL H5

TBI H4

TBI H1

CTL H1

TBI H2

CTL H2

TBI H3

CTL H3

CTL H4

TBI H5

2wk


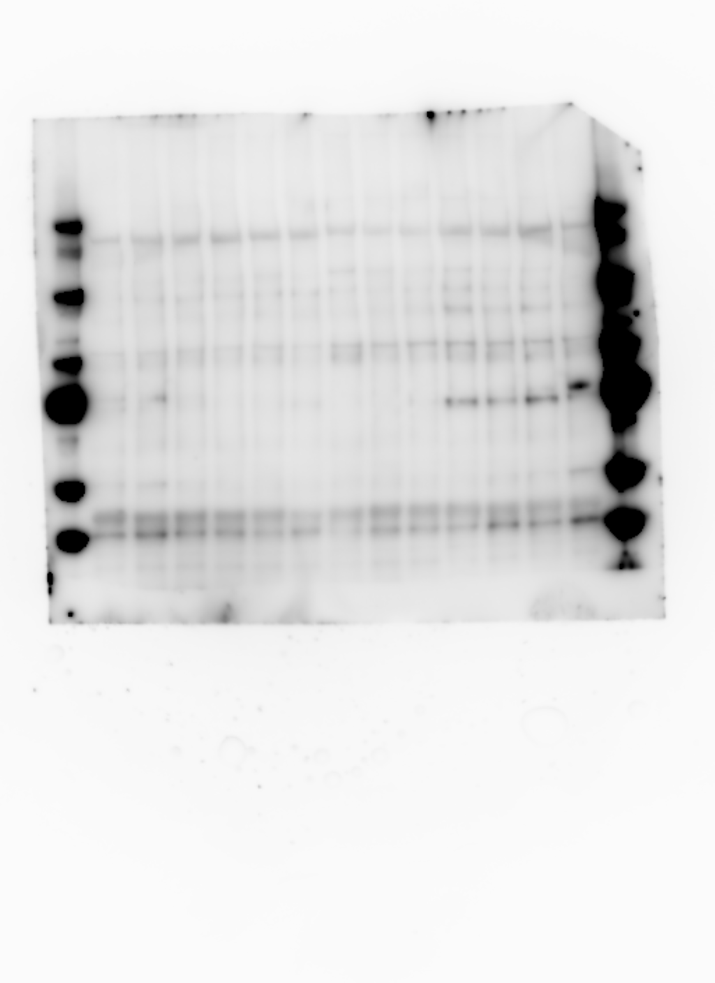


TBI H1

CTL H1

ladder

ladder

REF

CTL H6

CTL H5

CTL H4

CTL H3

CTL H2

TBI H6

TBI H5

TBI H4

TBI H3

TBI H2

4wk


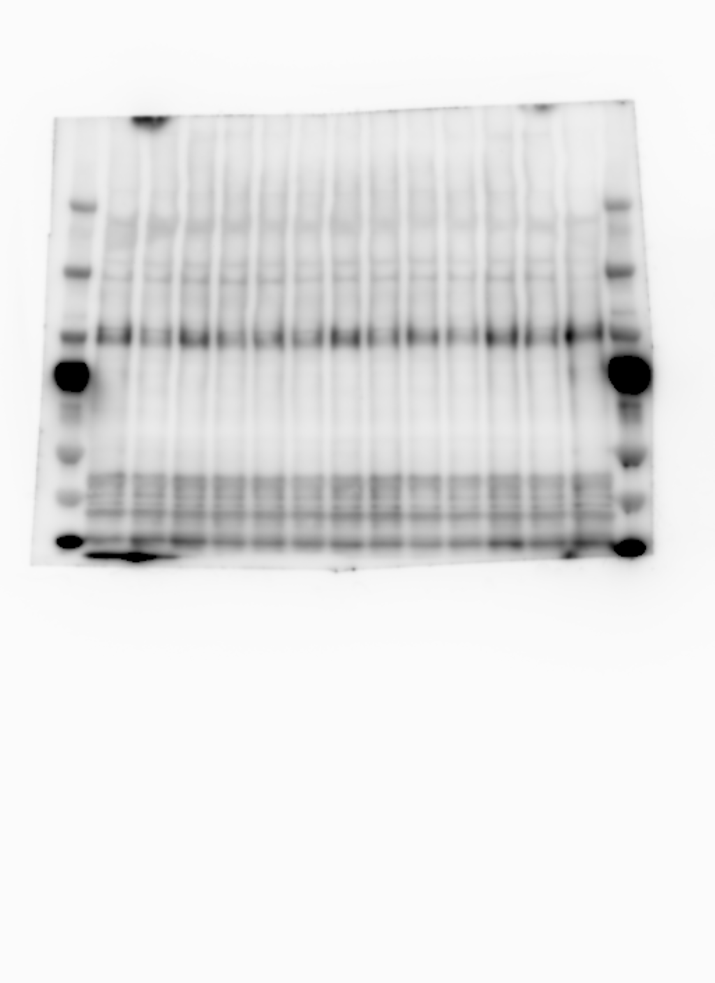


CTL H4

TBI H4

TBI H5

CTL H5

TBI H6

REF

ladder

CTL H6

CTL H2

TBI H2

CTL H1

TBI H1

TBI H3

CTL H3

ladder

**Figure 3C – lipids**

Cropped images in Figure are all lightened 1.25 exposure, unaltered images shown here

48hr


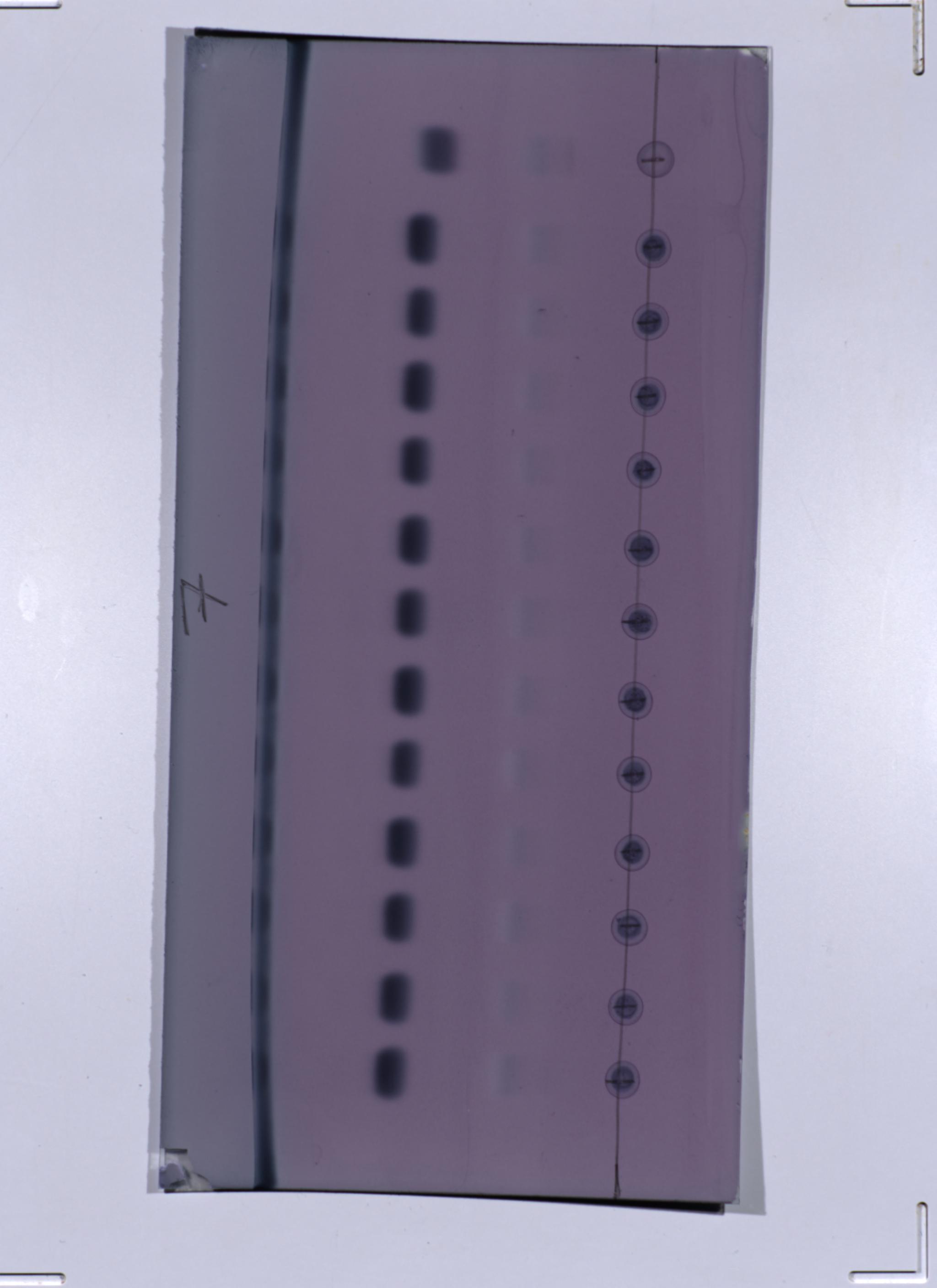


CTL 1

REF

CTL 6

TBI 6

CTL 5

TBI 5

CTL 4

TBI 4

CTL 3

TBI 3

CTL 2

TBI 2

TBI 1

2wk


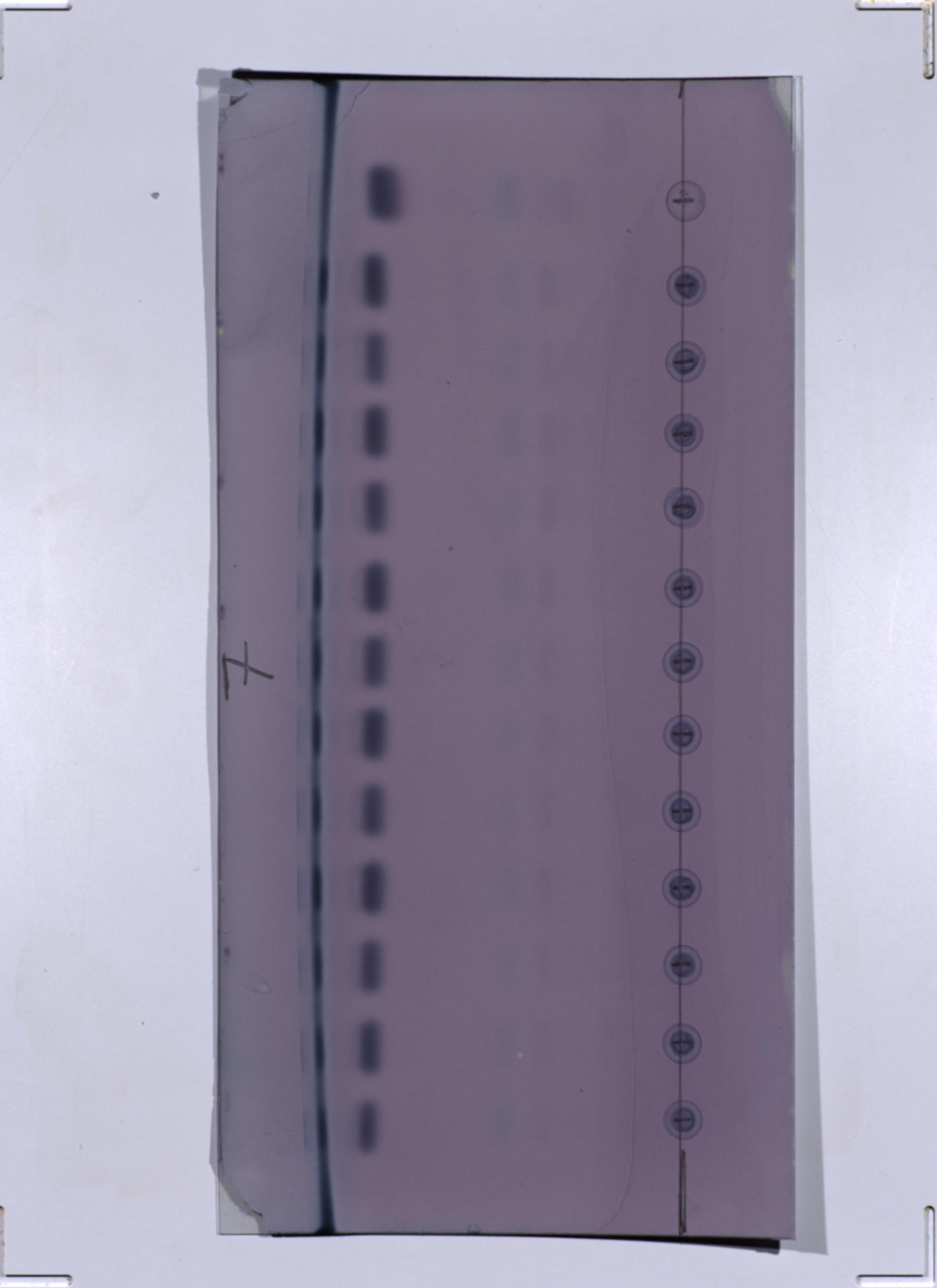


REF

TBI 1

TBI 2

TBI 3

TBI 4

TBI 5

TBI 6

CTL 1

CTL 2

CTL 3

CTL 4

CTL 5

CTL 6

4wk


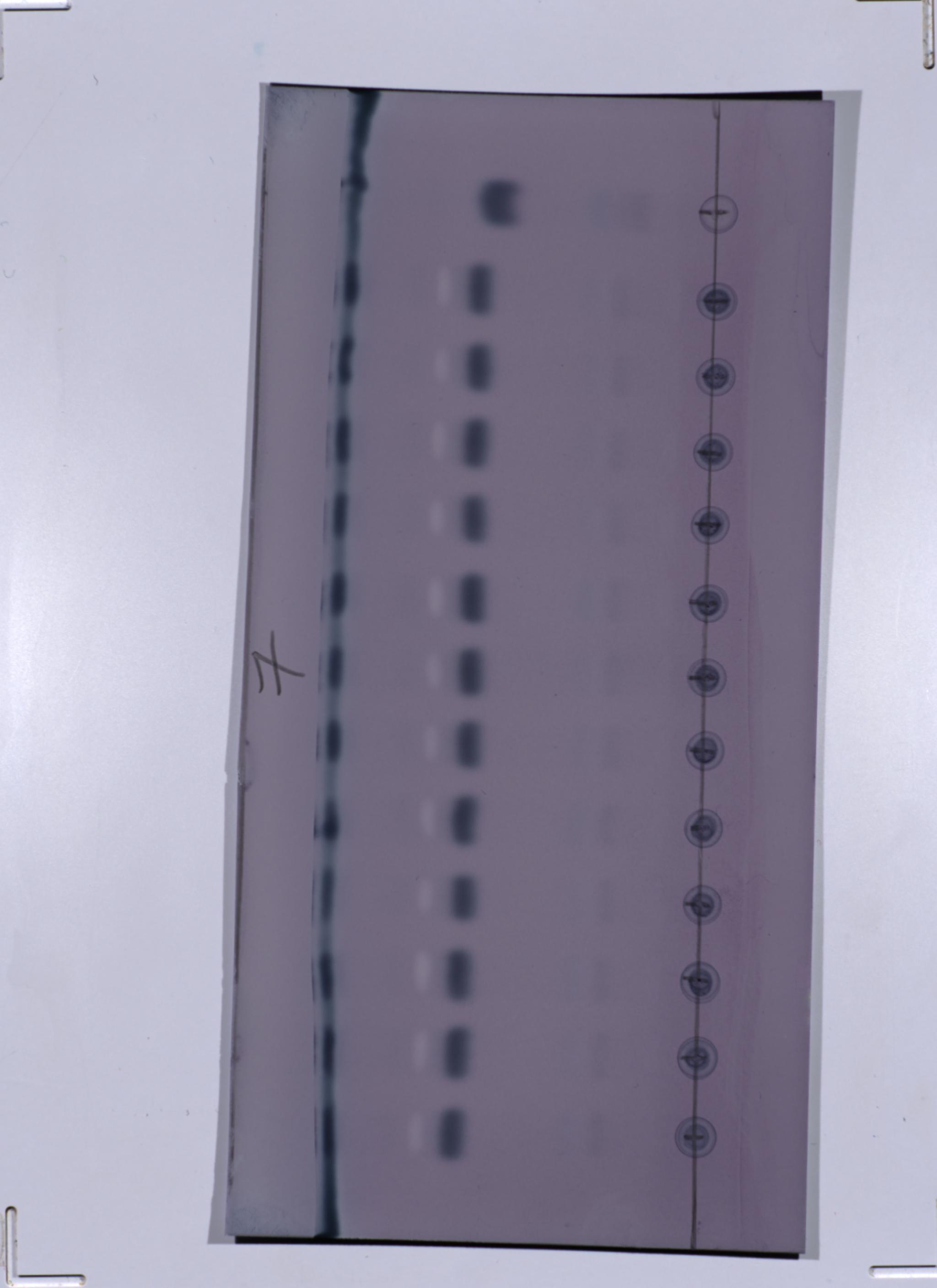


CTL 5

TBI 5

CTL 4

TBI 4

CTL 3

TBI 3

CTL 2

TBI 2

CTL 1

TBI 1

REF

TBI 6

CTL 6

**Figure 4E – Ubi heads**

6%

ladder

ladder

TBI H4


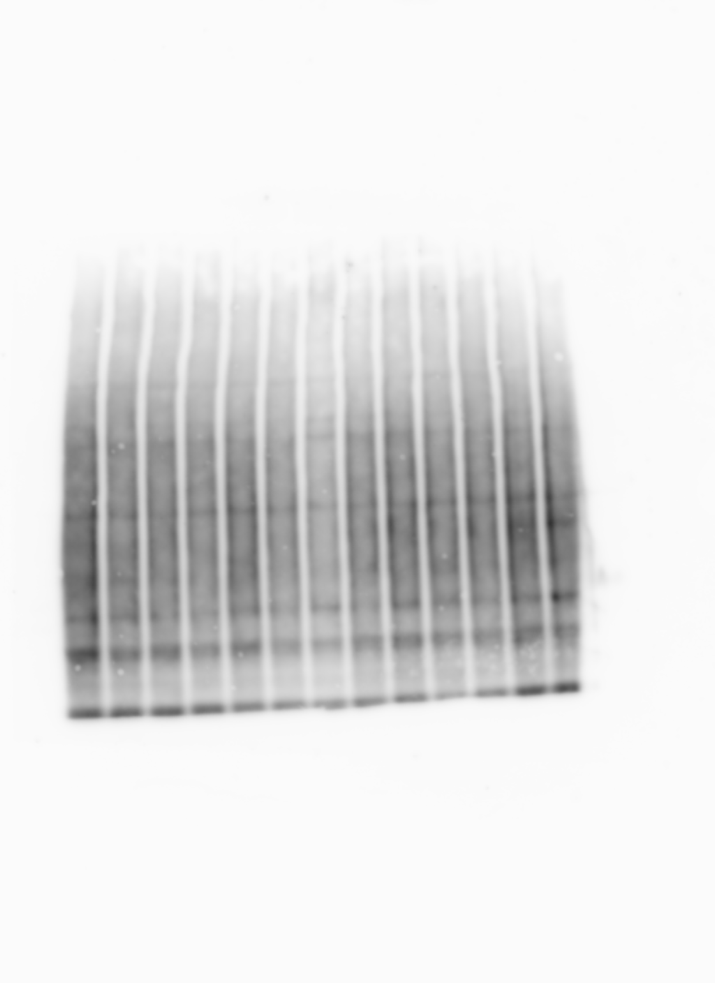


CTL H6

REF

TBI H1

CTL H1

TBI H2

CTL H2

TBI H3

CTL H3

CTL H4

TBI H5

CTL H5

TBI H6

1%


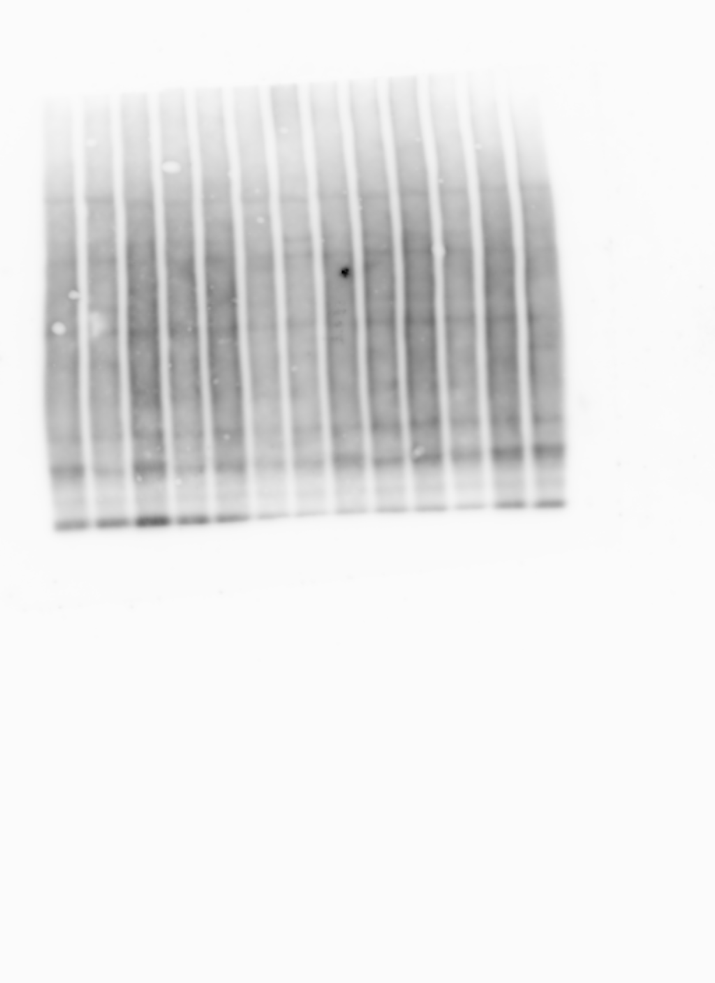


ladder

TBI H1

CTL H1

CTL H2

TBI H3

CTL H3

REF

TBI H4

CTL H4

TBI H5

CTL H5

TBI H6

CTL H6

ladder

TBI H2
